# Is neuroplasticity in the central nervous system the missing link to our understanding of chronic musculoskeletal disorders?

**DOI:** 10.1186/s12891-015-0480-y

**Published:** 2015-02-12

**Authors:** René Pelletier, Johanne Higgins, Daniel Bourbonnais

**Affiliations:** 1 grid.14848.310000000122923357École de réadaptation, Faculté de médecine, Université de Montréal, C.P. 6128, succursale Centre-ville, Montréal, H3C 3 J7 Québec Canada; 2 Centre for Interdisciplinary Research in Rehabilitation of Greater Montreal (CRIR), Institut de réadaptation Gingras-Lindsay-de-Montréal, Montréal, Québec Canada

**Keywords:** Musculoskeletal disorders, Chronic low back pain, Osteoarthritis, Neuroplasticity, Periaqueductal grey, Rostral ventromedial medulla, Rehabilitation, Primary somatosensory cortex, Primary motor cortex, Limbic, Pre-frontal, Pain

## Abstract

**Background:**

Musculoskeletal rehabilitative care and research have traditionally been guided by a structural pathology paradigm and directed their resources towards the structural, functional, and biological abnormalities located locally within the musculoskeletal system to understand and treat Musculoskeletal Disorders (MSD). However the structural pathology model does not adequately explain many of the clinical and experimental findings in subjects with chronic MSD and, more importantly, treatment guided by this paradigm fails to effectively treat many of these conditions.

**Discussion:**

Increasing evidence reveals structural and functional changes within the Central Nervous System (CNS) of people with chronic MSD that appear to play a prominent role in the pathophysiology of these disorders. These neuroplastic changes are reflective of adaptive neurophysiological processes occurring as the result of altered afferent stimuli including nociceptive and neuropathic transmission to spinal, subcortical and cortical areas with MSD that are initially beneficial but may persist in a chronic state, may be part and parcel in the pathophysiology of the condition and the development and maintenance of chronic signs and symptoms. Neuroplastic changes within different areas of the CNS may help to explain the transition from acute to chronic conditions, sensory-motor findings, perceptual disturbances, why some individuals continue to experience pain when no structural cause can be discerned, and why some fail to respond to conservative interventions in subjects with chronic MSD. We argue that a change in paradigm is necessary that integrates CNS changes associated with chronic MSD and that these findings are highly relevant for the design and implementation of rehabilitative interventions for this population.

**Summary:**

Recent findings suggest that a change in model and approach is required in the rehabilitation of chronic MSD that integrate the findings of neuroplastic changes across the CNS and are targeted by rehabilitative interventions. Effects of current interventions may be mediated through peripheral and central changes but may not specifically address all underlying neuroplastic changes in the CNS potentially associated with chronic MSD. Novel approaches to address these neuroplastic changes show promise and require further investigation to improve efficacy of currents approaches.

## Background

The treatment of Musculoskeletal Disorders (MSD) has been guided by a structural-pathology paradigm where the source of dysfunctions associated with the injury are to be found locally at the site of injury, the premise of “end organ dysfunction” [1]. The structural-pathology paradigm helps to comprehend and guide treatment effectively in acute MSD. There are however many unanswered questions and discrepant findings with chronic MSD where the structural-pathology paradigm fails as a working model for comprehension, research and in treatment. These include allusive questions such as why diagnostic findings correlate poorly with pain and dysfunction, the presence of bilateral findings with unilateral injuries, why a large proportion of persons with damage to musculoskeletal structures are asymptomatic, why some persons heal and others develop chronic MSD, and persisting sensory motor abnormalities [2-6]. In an attempt to better understand the clinical and experimental manifestations of these disorders researchers have expanded their scope of inquiry to include neurophysiological processes and plasticity within the Central Nervous System (CNS) associated with MSD.

Neuroplasticity is an intrinsic fundamental neurophysiological feature that refers to changes in structure, function and organisation within the nervous system that occurs continuously throughout a person’s lifetime [7-10]. Recent studies have revealed structural and functional changes within the CNS of people with chronic MSD. These changes are believed to be reflective of adaptive neurophysiological processes occurring with MSD that are initially beneficial and aid in the healing process by protecting the injured structures from further insult. In a chronic state, the structural pathology paradigm dictates that that these neuroplastic changes associated with chronic MSD are secondary to the injury and result from ongoing altered sensory transmission arising from the area of the musculoskeletal injury. Clinical and experiment findings however challenge this belief and demonstrate that neurophysiological adaptations may persist and be implicated in the development and maintenance of chronic signs and symptoms, possibly in lieu of healing to the peripheral musculoskeletal structures or co-existing with peripheral mechanisms [1,11]. It has recently been proposed that chronic pain associated with MSD is the result of imprinting, an implicit and/or explicit learned response that has formed a maladaptive memory sustaining the persistence of chronic pain [12-15]. According to this hypothesis, associative learning resulting from the initial trauma and subsequent events that reinforces the concurrent pairing between movement and pain results in an aversive association that is reflected and maintained by plastic changes in the meso-limbic and prefrontal areas [15].

This article will argue that neuroplastic adaptations and their effects may initially result from structural injury, but in chronic conditions contribute to the pathophysiology of the condition possibly even in the absence of any continued anatomical/structural insult to musculoskeletal structures. These neuroplastic changes explain many of the experimental and clinical findings present in subjects with chronic MSD. These changes result in sensory amplification [16], changes in sensory and motor representations [17-19] resulting in perceptual changes in body image [20,21], changes in motor control [22], bilateral experimental findings [23-25], the persistence and amplification of pain [16,26], and why some individuals transit from acute to chronic disorders [27,28]. Further evidence arguing to the importance of these neurophysiological adaptations stem from recent studies targeting neuronal processes appear to restore function and decrease pain [14,29,30]. These findings are highly relevant for the design and implementation of rehabilitative interventions for MSD which when guided by the structural-pathology paradigm have limited success in the treatment of many of these chronic conditions [31]. If neuroplastic changes in the CNS are not simply an epiphenomenon but are part and parcel to the pathophysiological process in chronic MSD, interventions that target these underlying pathophysiological mechanisms have the greatest chance of success [32]. Current conventional interventions in rehabilitation do not usually address underlying neuroplastic changes in the CNS associated with MSD [32] and the incapacity to effectively treat these chronic MSD stem as they are incomplete and/or misdirected [1,31,33-35].

## Discussion

The structural pathology paradigm is guided by the inherent belief that pain and other neurophysiological changes are secondary to local structural insult to musculoskeletal structures. Both in animal and human studies, it is apparent that local and systemic inflammatory responses, cellular and vascular proliferative changes as well as degeneration and fibrosis are all hallmarks of chronic and overuse MSD [34,36-41]. Injury to musculoskeletal structures, inflammatory mediators, and subsequent fibrosis change the mechanics of muscles and connective tissues affecting their physical properties and these in turn impact sensory receptor activity and transmission [11,34,42-46]. Under the structural-pathology paradigm neurophysiological consequences, with the exception of damage to the nerve(s), is secondary and should disappear when normal tissue properties are restored and receptor activity, sensory transmission, and perception should renormalize to reflect the state of the healed structure(s). Within this paradigm pain is simply a symptom and reflects the degree of damage to the musculoskeletal structure and associated biological responses locally in the area of injury. This viewpoint is supported by the findings that demonstrates the reversal of some, but not all Central Nervous System (CNS) changes when anatomical insult to musculoskeletal structures and pain disappears [47,48].

This paradigm however fails to explain many of the experimental findings with chronic MSD. For example, on a population level anatomical insult to musculoskeletal structures correlates poorly with diagnostic findings and these across a wide range of musculoskeletal disorders [2-6]. Therefore structural damage to musculoskeletal structures alone cannot always fully explain the presence of signs and symptoms in chronic MSD. Cognitive based interventions that involve education of pain processing and faulty beliefs regarding pain and movement yield better outcomes, between 10-20% improvement in disability and performance scales [49], than interventions involving education of anatomical and structural basis of injury [49-52] suggesting that central rather than peripheral influences play a key role in the clinical and experimental manifestation of at least some chronic MSD [51], and that clinical interventions aimed to modify the central processing of pain should be further evaluated and compared to clinical interventions targeting peripheral mechanisms.

### Principles of experience dependent plasticity

Neuroplasticity refers to changes in neuronal properties, structure and organization and is the manner in which the nervous system encodes new experiences. Neuroplastic changes have been demonstrated in response to experience and behaviour [53-56], motor learning [57-62], pain [17,63-65], injury [66,67], sensory stimuli [68-71], and cognitive processes [53,56,72,73]. Changes can be transient, reflecting the adaptability of the sensorimotor system to respond to internal and environmental demands and can occur over short training periods [74,75]. Neuroplastic changes in sensory-motor areas are stimulus driven and result in lasting neuroplastic changes when the internal and external pressures are repetitive, salient, involve learning and require sustained attention [7,53,54,76-78]. Neuroplastic changes have been observed in different areas of the CNS including the spinal cord, subcortical and cortical areas.

### Plasticity in the spinal cord and brain stem with chronic MSD

Sensory testing has demonstrated changes in sensory transmission and processing across a number of MSD including osteoarthritis (OA) [79,80], Patella-Femoral Pain Syndrome (PFPS) [81], tendinitis [82], Lateral Epicondylitis (LE) [83], Carpal Tunnel Syndrome (CTS) [84], lumbar [85] and cervical injuries including whiplash [86]. These studies include findings of changes in perception threshold to noxious and innocuous stimuli, but also other sensory alterations including stimuli being processed more slowly, incorrect localization, and decreased accuracy in recognition of tactile stimulation [43,79,81-85,87-92]. These changes have been demonstrated bilaterally and in sites remote to the initial injury [81,83,93]. Proprioceptive deficits include increased errors in repositioning [94-96], decreased position sense and ability to detect joint motion [97,98], difficulty to adopt postures seen on a photograph [87,89] across a number of MSD.

Although not all studies involving subjects with chronic MSD demonstrate altered sensory transmission [99] many studies with chronic MSD demonstrate augmented nociceptive transmission involving responsiveness to normally sub threshold nociceptive stimuli that results in hyperlagesia, an increase in nociceptive transmission and pain perception, indicative of an altered stimulus–response relationship to nociceptive stimuli, a process called Central Sensitization [16,26,83-85,100-102]. This is a normal, adaptive and reversible process that is biologically advantageous to protect the injured structure from further insult and is a consistent notion within the structural-pathology paradigm [26].

Neurophysiological changes also result in the amplification of noxious and innocuous stimuli within the dorsal horn of the spinal cord that persist in chronic pain states. These changes are reflective of processes similar to experience dependent plasticity and result from segmental, spinal and supraspinal processes that modulate membrane excitability and affect inhibitory and facilitatory processes within the spinal cord (see [16]). Some dorsal horn nociceptive neurons develop increased receptor field size (wide-dynamic range neurons) responding to nociceptive and cutaneous stimuli that results in secondary hyperalgesia and allodynia (spread and perception of pain with innocuous stimulation) [16].

The supraspinal influences on dorsal horn nociceptive transmission include descending pain modulatory systems including the Periaqueductal grey (PAG)-Rostral Ventromedial (RVM) pathway. Under normal circumstances these systems inhibit the transmission of nociceptive stimuli in the dorsal horn of the spinal cord [103]. There exists convincing evidence in animal models that these descending modulatory systems are disrupted in chronic pain subjects shifting from a state of inhibition to a mal-adaptive state of facilitation amplifying the transmission of nociceptive stimuli, contributing to the process of central sensitization, and perpetuating the augmented transmission of neuropathic stimuli [16,45,103]. For example, an increase in activity of cells that project to the dorsal horn of the spinal cord from the RVM that facilitate the transmission of noxious stimuli is present only in animals with neuropathic pain behaviours [104]. The microinjection of lidocaine into the RVM, causing a temporary cessation of neuronal activity, and an ipsilateral lesion of the dorsal lateral funiculus that house neuronal projections from the RVM towards the dorsal horn both decrease the threshold to elicit withdrawal reflexes, indicative of increased pain perception and that neuronal activity of the RVM is facilitating the transmission of nociceptive/neuropathic stimuli [105]. Electrical stimulation of the RVM paired with cutaneous stimulation recorded from second order spinal nociceptive neurons results in a 130% increase in neuronal activity [106]. In CLBP patients there is a decrease in PAG cerebral blood flow not seen in healthy control subjects suggestive of decreased neuronal activity [107]. In humans there is evidence that the a test noxious stimulus, under normal circumstances, is inhibited by a preceding noxious conditioning stimulus, a process called Conditioned Pain Modulation [108], and is disturbed in subjects in some MSD and chronic pain states [108,109]. Collectively the results from these studies demonstrate that the PAG-RVM pathway not only facilitates nociceptive transmission in the dorsal horn of the spinal cord but actually perpetuates the transmission of pain. This argues against a peripherally driven source of augmented nociceptive/neuropathic transmission and for a centrally mediated mechanism perpetuating the transmission of afferent stimuli that is inconsistent with the structural-pathology paradigm.

Neuroplastic changes amplifying sensory transmission have functional implications. Subjects demonstrating central sensitization, hypersensitivity and allodynia have a poorer prognosis to treatment including surgical interventions for varied MSD [12,100,110,111]. Furthermore, studies in both animals and humans demonstrate that altered sensory transmission may result in changes in neuronal properties and organization within different subcortical and cortical areas including the thalamus, primary somatosensory cortex (S1) and the primary motor cortex (M1) implicated in sensory transmission, perception and motor control [112,113].

### Neuroplastic changes in the primary somatosensory cortex and perceptual changes with MSD

Studies of cortical properties and organisation within the sensorimotor areas have been performed with subjects with PFPS [114], anterior cruciate ligament (ACL) deficiency and reconstruction [33,115-117], CLBP [17-19,118-121], cervical pain and whiplash injury [91,122], rotator cuff tears [123,124], dystonia [125-129] and CTS [130-133]. These studies suggest that neuronal properties, organization, and morphometric changes are present in subjects with chronic MSD. For example, subjects with CLBP demonstrate a 2.5 cm shift of the somatotopic representation in S1 [17,121] and grey matter volume changes that correlate with chronicity of symptoms [134,135]. Studies in subjects with CTS reveal changes along the afferent pathway in the spinal cord, brain stem and S1 [131], a decrease in grey matter volume [133] and a loss of spatially segregated representations of digits 2 and digits 3 in the contralateral S1 that correlate with changes in nerve conduction velocity [131,133,136]. Somatotopic re-organisation in CTS subjects are specific to the nature of sensory stimuli as the representation of the digits in S1 is decreased with pain and increased with paraesthesia [130].

In the perspective of the structural-pathology paradigm, these changes in S1 associated with MSD may simply be reflective of altered peripheral sensory transmission reflective of altered afferent peripheral sensory stimuli and transmission occurring as the result of insult to musculoskeletal structures and inflammation. Studies in non-human primates with peripheral de-afferentation and spinal cord injury demonstrate degeneration in the cuneate nucleus of the brainstem, an area that contains axons from the dorsal root ganglion transmitting cutaneous and proprioceptive stimuli, as well as somatotopic reorganization in an area of the thalamus (ventral posterior lateral nucleus) that transmits sensory afferent stimuli to S1. The changes in S1 in these studies mirror the changes found in the thalamus suggesting that the changes in sensory afference including noxious, cutaneous, and possibly proprioceptive afferent transmission are implicated in S1 reorganization [112,113]. However, should altered afferent transmission persist, potentiated by functional changes in the brain stem and the spinal cord, neurophysiological changes appear to result in behavioural and functional implications that are not simply a reflection of altered sensory afference.

There is growing evidence that pain associated with MSD such as osteoarthritis and CLBP may be, at least in part, the result of the plasticity of the sensory representation of the body and perceptual disturbances [137-139]. Distortions in body image have been found in a range of conditions where cortical reorganization in S1 are present including Phantom Limb Pain (PLP), Complex Regional Pain Syndrome (CRPS) and in CLBP [14,20,140-142]. These changes include the sensation of abnormal size, shape, swelling, and position [30]. Perceptual changes may also arise from abnormal or conflicting sensory and/or motor inputs [143,144]. Perceptual changes also have functional implications. Incongruence and manipulation between sensory and motor input has been shown to cause sensory disturbances, and aggravate symptoms and pain [145]. Modulation of the shape and size of a limb can impact tactile acuity and pain [146]. Visual distortion of the hands in subjects with osteoarthritis helps to decrease pain [137]. Interventions targeting changes in somatotopic reorganization through the use of sensory discriminative training and visual distortion can renormalize the S1 representation and decrease pain [30,139,147-149]. The modulation of the size of the limb can alter subjective feelings of pain and motor imagery can cause an increase in pain and swelling that cannot be attributed to increased peripheral sensory afference arising from nociceptors or peripheral neural injury [142,150]. The persistence of abnormal motor imagery in recurrent low back subjects is also believed to be reflective of ongoing disruption of cortical maps even in the absence of pain [151]. These findings support the belief that structural injury to musculoskeletal structures are not the only driver of pain and dysfunction, CNS changes play an active role in the pathophysiology of chronic pain conditions, and interventions that target these CNS changes may decrease pain, improve function, and even affect mechanisms involved in the local biological response to injured structures such as swelling.

### Changes in primary motor cortex associated with MSD

Studies that investigate changes in the properties, function and organisation within the primary motor cortex (M1) of subjects with different MSD have been performed, of which the majority utilise Transcranial Magnetic Stimulation (TMS). TMS produces a high intensity electrical pulse resulting in a magnetic field perpendicular to the stimulating coil. The magnetic pulse traverses the skull and when applied over the motor cortex with sufficient intensity, can depolarize corticospinal neurons directly or indirectly. This stimulation results in the depolarization of different motoneuron pools within the spinal cord and an electromyographic response, the Motor Evoked Potential (MEP) can be recorded. Utilising different parameters of stimulation and experimental protocols, TMS allows for the appreciation of corticospinal excitability, inhibitory and facilitatory processes, and somatotopic organization of corticospinal neurons. Studies of corticospinal excitability have been performed in subjects with various MSD including PFPS [114], ACL deficiency [117], CLBP [18,19,119,120,152,153], and Rotator Cuff Tears [123,124]. Collectively these studies demonstrate changes in corticospinal excitability that correlate with pain and disability scores. Changes in motor behaviour that are present in subjects with CMSD appear to be largely mediated by changes in the cortical areas including M1. Inhibition of corticospinal output is increased in experimentally induced muscle pain resulting in decreased motor responses to TMS at rest [154] and increased corticospinal output during forceful muscle contractions [155,156]. Findings from these studies appear to be consistent with the experimental findings that demonstrate variable motor control changes including reorganization of motor unit recruitment both within and between muscles in an attempt to minimize the motor consequences associated with chronic MSD (see [11,22,65]), co-activation of muscles and overlapping of muscle/movement representations in M1 [19,22], and variations in corticospinal output in an attempt to maintain constant force under painful conditions and compensate for increased inhibition [155].

In a series of experiments Tsao and his colleagues investigated the properties and organization of the representation of muscles in the lumbar spine within M1 in subjects with CLBP. They demonstrated that the area of corticospinal recruitment of muscles of the lumbar spine in M1 is altered in CLBP subjects [18]. These changes correlate with changes in motor recruitment [18,19]. Motor skill learning involving exercises to specifically recruit the transverse abdominus muscle, but not a walking exercise, could restore the representation within M1 and EMG activation pattern in CLBP subjects to that seen in healthy controls [118]. The changes in the representation of the movements elicited by the trunk muscles in M1 are associated with the impaired activation of these muscles and may underpin changes in motor activation, specifically the inability to selectively recruit these muscles. This, in turn is consistent with the increased activation of superficial muscles in this population when performing movements [157] and the altered activation of the multifidus that has been demonstrated in patients with recurrent LBP [158,159]. These studies demonstrate that neuronal properties and organisation within M1 are modified in CLBP subjects and that intervention specifically targeting these representational changes improve function and decrease pain.

The relationship between the plastic changes in the spinal cord, brain stem and cortical sensori-motor areas are complex. Experimental findings suggest the possibility of two-way causality, where altered sensory input including enhanced nociceptive/neuropathic stimuli, altered cutaneous and proprioceptive input affects sensorimotor organisation and processes within the CNS, and these changes in turn affect perception, pain, and motor control processes contributing to the pathophysiology of the condition [137,138]. If these processes remain present for a substantial period of time they may result in lasting neurophysiological adaptations that may become imprinted and can outlive the insult to peripheral musculoskeletal structures [14,15]. It is important to note that a return to before injury sensory transmission and the performance of repetitive strengthening exercises may not be sufficient to return the neuronal properties and organization within the sensorimotor areas to a pre-injury state [160]. Specific interventions addressing these neuroplastic changes in sensorimotor areas appear to be required. Repetitive unskilled movements do not result in neuroplastic changes in M1 [57,76]. Motor skill training however has proven successful in the treatment of some musculoskeletal conditions, improves task performance and helps promote neuroplastic changes in M1 [53,118,161-165]. These findings are suggestive that the neuroplastic changes in the sensory-motor areas are implicated in the pathophysiology of some chronic MSD and should impact rehabilitative treatments.

### Role of pain in CNS plasticity

Findings from experimental studies do provide convincing evidence that pain provides an impetus for CNS changes with MSD. Experimentally induced pain impacts neuronal properties and organisation in S1 and M1 [153,166] and subjects with chronic pain associated with unilateral herpes simplex virus have a decreased representation between digits 1–5 in the contralateral S1 [167]. Although the causal relationship between pain and cortical reorganization has not been definitively established with MSD, the evidence suggests that pain is a driver of cortical re-organization. In other conditions where re-organisation in S1 is present there is a renormalisation with the attenuation of pain [168,169] and some, but not all, of the morphological changes in brain grey matter volume and changes in cortical somatotopy return to those seen in normal healthy subjects when pain is eliminated [47,48,168,169].

However pain alone is neither necessary nor sufficient to drive neuroplastic changes. Dystonia and CTS are both conditions where researchers have demonstrated neuroplastic changes in M1 and S1 in the absence of pain. Focal hand dystonia involves a loss of individual control of the digits of the hand that results from rapid repetitive motor actions of the fingers. These movements result in blurring of the representation of the digits with loss of spatial segregation [127-129]. Subjects with recurrent low back pain continue to demonstrate abnormal motor control in the absence of pain possibly reflecting continued reorganisation of neuronal properties and organisation in M1 [170-172]. Behavioural interventions that help to restore somatotopic organisation also improve function and decrease pain suggesting the possibility of two way causality between pain and sensorimotor representations [173].

Although pain provides an impetus for neuroplastic changes in the CNS, other forms of stimuli, cognitive processes and behaviours can induce plastic changes. Studies in animals, healthy human and neurologically compromised human subjects have demonstrated that repetition and attention/salience are important factors inducing neuroplastic changes in S1 and M1 [7,77,174,175]. The limbic and prefrontal structures are the cortical areas responsible for these aspects of behaviour and findings have demonstrated important changes in these areas in chronic pain states including some MSD [13,15].

### Neuroplastic changes in meso-limbic and prefrontal structures in chronic pain states

Of all the areas of the CNS with documented changes occurring in association with chronic MSD, the meso-limbic and prefrontal structures are the most impressive and possibly the most important as changes in these areas demonstrate strong correlations with chronicity [13], and furthermore can be predictive and possibly even determine who will transit from acute to chronic pain [15,27,28]. Experimentally induced pain results in the activation of characteristic cortical regions including S1, S2, insula, cingulate cortex, amygdala, and prefrontal cortex in what is commonly referred to as the pain matrix, but is possibly more reflective of a salience network as these structures are not only active with painful stimuli but also in conditions involving increased attention/salience [176,177].

Experimental findings suggest that the structure and function of the brains of subjects with chronic pain including CLBP and OA are different from healthy controls and this is most important in the meso-limbic and prefrontal areas (see [13]). When experimentally induced pain is applied to subjects with CLBP and osteoarthritis (OA) while performing a fMRI, both CLBP and OA subjects demonstrate spontaneous fluctuations of pain that is not time locked to the experimental noxious stimuli and are not present in healthy control subjects [178,179]. Spontaneous pain engages pre-frontal and limbic areas important for the processing and cognitive response to incoming stimuli [13,178,180]. FMRI studies have demonstrated that subjects with chronic MSD, specifically CLBP and OA, demonstrate abnormal activity in the cingulate cortex, the amygdala, the insula, nucleus accumbens (NAc) and pre-frontal areas including the medial prefrontal cortex (mPFC) and the dorsolateral prefrontal cortex (dlPFC) [13,134,178]. These mesolimbic-prefrontal areas are involved in the cognitive affective aspects of pain and injury including the behavioural response to these, the processing of fear, emotions, negative conditioning and attention [181,182]. One result of the abnormal activity in these areas is increased vigilance and a decreased ability to disengage from pain [12]. These limbic structures have direct and indirect connections with both the sensorimotor areas and the brain stem and may provide the substrate of attention and salience necessary for the induction of neuroplastic changes in these areas [180,183]. Furthermore, these structures influence descending pain modulatory systems including the PAG-RVM pathway where, as discussed earlier, compelling evidence suggests is disrupted in chronic pain subjects and perpetuate the ongoing abnormal augmented pain transmission originating from nociceptive and non-nociceptive peripheral receptors in the dorsal horn of the spinal cord [103,184].

The brain derived biomarkers from abnormal activity in the mesolimbic and prefrontal areas correlate strongly with clinical measures in patients with CLBP and correlate better with clinical findings than do structural and psychosocial findings [13,184]. Increased insular activation is correlated with pain duration, while mPFC activation is correlated with pain intensity in CLBP subjects [13]. Abnormal increased connectivity between the mPFC and the NAc is highly predictive (90%) of who will go on to develop CLBP suggesting that there may be pre-disposing biomarkers for the development of chronicity [28,179]. For a more thorough overview of changes in the meso-limbic and prefrontal areas associated with CMSD excellent reviews have been published [13,15,184].

The complex interrelationship between pain, cortical reorganization, disability, and abnormal motor behaviour is compounded by the implication of psychological factors associated with chronic pain and injury. Catastrophization (“tendency to focus and magnify pain sensation, and to feel helpless in the face of pain”) and fear play a role in the etiology and prognosis of chronic pain conditions [185-188]. Psychosocial factors predict variance in pain, gait velocity, and psychological disability in OA subjects, appear to increase pain and disability (see [185,186]), impact pain perception in healthy controls [186,189], and may result in a learned avoidance behaviour perpetuating the disability [185,190]. These changes in the pre-frontal cortex activation are also consistent with fMRI studies that have correlated changes in prefrontal activity with psychosocial variables involved in CLBP and OA, including dlPFC activity being negatively correlated with Pain Catastrophizing Scores and mPFC activity correlated with fear-avoidance/anxiety [12,191,192]. Pain catastrophizing and fear-avoidance cause behavioural changes and may be responsible for changes in neuronal properties and somatotopic reorganization because of disuse similar to learned non-use in stroke patients [193,194]. Neural circuits not actively engaged in task performance for an extended period of time begin to degrade [7,195]. Prolonged non-use of the affected limb may lead to a vicious cycle whereby immobility, changes in cortical representation, and atrophic changes re-enforce each other.

### Integrating CNS changes into a more comprehensive model of chronic MSD

It would appear that behavioural changes and psychological processes in chronic pain subjects involve activity in the meso-limbic and pre-frontal areas that influence pain perception and behaviour. Although speculative, the behavioural changes associated with these changes in meso-limbic and pre-frontal areas may therefore be reflective of salience and increased attention directed towards the injury and associated pain. The meso-limbic and prefrontal structures influence descending modulatory pathways and facilitate the transmission of noxious stimuli which perpetuates the altered transmission of sensory stimuli and appear to influence sensorimotor representations and neuronal properties. It is possible that these changes collectively result in a vicious cycle where injury, pain, altered sensory transmission, sensorimotor changes, behavioural changes, salience, attention, and fear-avoidance may feed off one another perpetuating the disability. It has been hypothesized that the neuroplastic cortical changes in the meso-limbic prefrontal areas associated with chronic pain states are reflective of learned operant and classic conditioning resulting in the formation of a “pain” memory [12-14,196]. Providing support for this hypothesis are findings where imagery affects pain, swelling, and cortical excitability [150]. Consistent with the implication of altered neuronal activity in the meso-limbic and prefrontal areas in the pathophysiology of chronic MSD are the findings from educational and cognitive based interventions. Educational programs explaining the neurophysiological mechanisms of pain have proven more effective than back schools (which emphasize end organ dysfunction and behavioural changes to decrease loading of anatomical structures) in CLBP patients [49-51,197]. These educational programs attack faulty pain beliefs which lead to fear-avoidance often present with chronic MSD [198]. The findings that altered functional connectivity in these areas are the best predictors of chronicity in the transition from acute to CLBP further supports the argument as to the importance of the changes in these areas in the pathophysiology of MSD [28]. These findings are inconsistent with a structural-pathology paradigm of a solely peripherally driven source of dysfunction in chronic MSD. Chronic MSD such as OA and CLBP, and possibly other MSD may have prominent CNS contributions with peripheral and central factors, cortical and limbic areas, all playing a role in the pain and dysfunction they produce [11,45]. Collectively these findings of changes in meso-limbic and prefrontal structures provide compelling evidence that CNS changes contribute to the pathophysiology of at least some chronic MSD and conversely, that the structural-pathology paradigm of local tissue compromise being solely at the root of chronic MSD is at the very least incomplete and insufficient. A model integrating central neurophysiological modifications must be integrated into the present paradigm to broaden its scope and be further investigated.

### Impact of CNS plasticity in the rehabilitation of chronic MSD

Restoration of motor activity and function are integral to current practice in rehabilitation [51,199]. The notion of addressing neuroplastic changes is well established in neurological rehabilitation [32]. Interventions presently utilized in conventional rehabilitative care may result from peripheral and central mechanisms and it remains a challenge to distinguish their relative contribution. For example, resistance training in subjects with non-specific shoulder and neck pain increased local and distal pressure pain thresholds suggestive a central mechanism underlying these effects [200]. However studies also demonstrate that specific types of interventions may be better suited at inducing neuroplastic changes [10,62,76,160]. Rehabilitative interventions specifically addressing neurophysiological changes, in addition to peripheral end organ dysfunction, may prove to be an important avenue of investigation in the hope to improve treatment success in the rehabilitation of musculoskeletal injuries [10,32]. Studies in animal models have demonstrated that the neuroplastic changes in S1 and M1 occur concurrently with tissue damage, inflammation, and motor impairment and therefore would need to be addressed early on in the rehabilitation process [34,201]. Addressing neurophysiological changes would involve interventions in an attempt to minimize and/or normalize structure, function and organization to that found in uninjured healthy controls by explicitly targeting and priming neuronal structures and processes including those in the sensorimotor, meso-limbic and pre-frontal areas. These could include incorporating approaches to present conventional care such as education of neuronal and pain processes [51,197], cognitive based interventions such as Cognitive Behavioural Therapy [202,203] and Mindfulness Based Stress Reduction [204,205] which have been associated with changes in pre-frontal and meso-limbic structures [206-211], mental imagery [29], peripheral sensory and electrical stimulation [63,147], visual distortion and the use of non-invasive brain stimulation such as Transcranial Direct Current Stimulation and TMS for example to alter neuronal processes [212-214]. Effect sizes of rehabilitation approaches are consistently small regardless of intervention in many MSD and therefore multiple and progressive interventions may be warranted [51].

### Research

Research investigating changes in S1 and M1 across a large range of MSD, including changes in responsiveness, inhibitory processes, and somatotopic organization would help elucidate the mechanisms and their presence in MSD. Subsequent studies evaluating novel treatment approaches such as motor skill training, mental imagery, action observation, mirror therapy, peripheral sensory stimulation and cortical stimulation as adjuncts to traditional rehabilitative care for MSD to impact neuronal responsiveness and reorganization are needed. Research in changes in neuronal processes and organization of techniques presently utilized in rehabilitation, such as manual therapies, may help elucidate the physiological mechanisms of action and lead to more effective application and outcomes. Further research of the plastic changes occurring in meso-limbic and prefrontal areas and the complex interrelationship between structures and connections on these areas, cortical sensorimotor areas, descending modulatory processes, and psychological traits and behaviours associated with CMSD will not only increase our comprehension, but help guide the development of more effective pharmacological, behavioural and rehabilitative interventions.

## Summary

In our opinion the present structural-pathology paradigm guiding treatment for MSD is at the very least incomplete as it fails to integrate recent findings of important neurophysiological changes associated with chronic MSD and that appear to be involved in the pathophysiology of these conditions either in isolation or co-existing with peripheral mechanisms. Musculoskeletal injury, in addition to the local damage to anatomical structures and inflammation, results in changes in sensory stimuli, transmission and processing including neuroplastic changes along the neuroaxis of pain within the spinal cord and brain stem, in the properties and functions of neurons within S1 and M1. There are associated changes also found in the meso-limbic pre-frontal areas in subjects with chronic MSD some which may pre-dispose the injury. The neuroplastic changes may occur rapidly in response to injury causing adaptive changes that may help in the protection and healing response. However, these changes may persist and no longer perform their intended function contributing to the development of chronic disability and dysfunctional pain with enduring neuroplastic changes along the neuroaxis of pain resulting in peripheral and central sensitization, in the sensorimotor areas affecting perception and motor behavior, and in the meso-limbic prefrontal areas influencing emotional, attentional and cognitive processes [11,31,44]. In some musculoskeletal conditions the responsiveness and somatotopic organization in S1 and M1, including changes in excitability, the blurring of the representation of anatomical structures and a shift in the representation of muscles within somatotopic representations are present. These changes in properties, function and organization within the CNS often correlate with the severity and duration of pain, functional changes including aspects of motor control, psychological traits associated with the chronic pain states, and can be predictive of prognosis. These findings have important implications in the rehabilitation of MSD. Many questions remain to be answered including the specific nature of the contribution of these neuroplastic changes to the clinical condition specifically in relation to causation and how widespread these changes are with different MSD. In this respect, we are in agreement with the hypothesis that failure of rehabilitative and medical interventions to treat these chronic musculoskeletal conditions effectively may stem from failure to address these neuroplastic cortical changes and are of the opinion that the elaboration and evaluation of rehabilitative interventions, some presently utilised in neurological rehabilitation, in the prevention and treatment of chronic MSD are desirable [31,32].

## Abbreviations

MSD: Musculoskeletal disorders
CNS: Central nervous system
SEP: Somatosensory evoked potentials
CLBP: Chronic low back pain
OA: Osteoarthritis
PFPS: Patella-femoral pain syndrome
LA: Lateral epicondylitis
CTS: Carpal tunnel syndrome
PAG: Periaqueductal gray
RVM: Rostral ventromedial
S1: Primary somatosensory cortex
M1: Primary motor cortex
ACL: Anterior cruciate ligament
fMRI: Functional magnetic resonance imaging
SEP: Somatosensory evoked potentials
PLP: Phantom limb pain
CRPS: Complex regional pain syndrome
TMS: Transcranial magnetic stimulation
MEP: Motor evoked potential
NAc: Nucleus accumbens
mPFC: Medial prefrontal cortex
dlPFC: Dorsolateral prefrontal cortex
